# Three Optima of Thermoelectric Conversion: Insights from the Constant Property Model

**DOI:** 10.3390/e27030252

**Published:** 2025-02-27

**Authors:** Paul Raux, Christophe Goupil, Gatien Verley

**Affiliations:** 1 IJCLab, CNRS/IN2P3, Université Paris-Saclay, 91405 Orsay, France; 2 LIED, CNRS, Université Paris Cité, 75013 Paris, France

**Keywords:** thermoelectricity, energy conversion, optimisation

## Abstract

Starting from Ioffe’s description of a thermoelectric converter, we recover the optimal working points of conversion: the point of maximum efficiency and the one of maximal power. Inspired by biological converters’ optimization, we compute a third optimal point associated with cost of energy (COE). This alternative cost function corresponds to the amount of heat exchanged with the cold reservoir per unit of electric current used. This work emphasizes the symmetry between the efficiency and performance coefficient of the electric generator and heat pump modes. It also reveals the relation between their optimal working points.

## 1. Introduction

Optimizing the operation of thermodynamic machines is a very old subject, and in fact, one that dates back to the machine’s very beginnings. For a heat engine, Carnot’s efficiency is the maximum achievable efficiency of a heat-to-work conversion. It is an upper bound imposed by the second law of thermodynamics [1,2]. Although fundamental, because it only involves the thermostat’s temperatures, it requires the reversible transformation of the thermodynamic fluid in the converter. This situation is associated with null power output. On the contrary, the maximum efficiency achievable by an irreversible converter (with finite output power) is not universal. As usual with irreversible thermodynamics, some assumptions are required on the modeling of the converter [3,4]. Besides the maximum achievable efficiency, one can alternatively maximize the output power of a given converter. This situation is relevant for peak consumption with given infrastructures or when economic arguments come into play. For endoreversible converters, i.e., reversible converters imperfectly coupled to the thermostats through finite thermal conductivities, Curzon and Alhborn obtained the efficiency at maximal output power [5,6,7,8,9]. Like Carnot’s result, this efficiency at maximum power only involves the thermostat’s temperatures. However, here again, some assumptions are required on the irreversible thermodynamic modeling of the converter [10,11]. A third optimum is less common in physical science and appeared first for biological systems in which waste production may compromise homeostasis; it corresponds to minimal waste production per unit of biological useful flux [12,13,14].

In the present work, we follow up on the literature on the optimization of thermoelectric conversion using the linear irreversible thermodynamics framework [15,16,17,18,19,20,21]. We focus on the model of thermoelectric converter (TEC) called the constant property model (CPM) [22]. In a systematic way, we present the different operating modes, the conversion efficiencies, the optimal working points (maximum power or efficiency and minimal waste), and the trade-off between power and efficiency [23]. Our main aim is to provide a method for optimizing all the modes of operation of the TEC by only optimizing the electric generator (EG) efficiency. This is possible since the efficiencies in the heat pump (HP) modes are simple functions of the EG efficiency.

This study is outlined as follows: In Section 2, we define the partial entropy production rates (EPRs) associated with heat and work exchanges that are relevant for the two operating modes of the TEC:

(EG) electric generator mode,
(HHP/CHP) heating/cooling heat pump mode,

Below, the equations are labeled according to these acronyms to specify the operating mode they hold for. We emphasize that two utilities exist for the HP mode (cooling or heating). For all modes and utilities, we determine the range of compatible electric current. The associated device’s performance can all be expressed in terms of the EG’s efficiency. Accordingly, for fixed temperature difference at the boundary of the TEC and adjustable electric current, we study the optimal working points and the power-efficiency trade-off for the EG in Section 3 to draw conclusions on the optimal working conditions of the heating or cooling HP in Section 4.

## 2. Partial EPR, Operating Modes, and Efficiency

**Figure 1:**
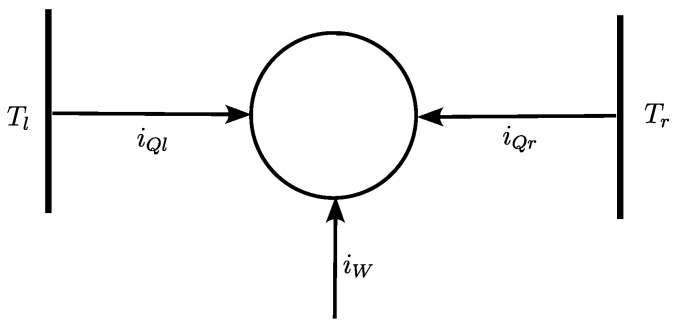
Schematic of the TEC: currents are positive if flowing in the direction of arrows. To fix the idea, we use $T_{l}>T_{r}$ and heat flows spontaneously from left to right thermostats in EG mode, involving a convective part due to electric current and a conductive part associated to heat loss.

Through convention, heat and work currents are algebraic, with a positive current chosen for those entering the system, e.g., the heat current dumped into the cold reservoir at $T_{r}$ is negative, see Figure 1. We use heat and work currents given as quadratic functions of the electric current $I_{C}$ (chosen as positive when flowing from the left to the right side of the thermoelectric material). With those conventions, the heat currents received from the left and right reservoirs and the electric power received by the TEC read as follows:

(1) $$\begin{array}{ccc} i_{Ql} & = & -K\Delta T+\left(\alpha T_{l}-\frac{R}{2}I_{C}\right)I_{C}, \end{array}$$

(2) $$\begin{array}{ccc} i_{Qr} & = & K\Delta T-\left(\alpha T_{r}+\frac{R}{2}I_{C}\right)I_{C}, \end{array}$$

(3) $$\begin{array}{ccc} i_{W} & = & -I_{C}\Delta V=\left(\alpha \Delta T+RI_{C}\right)I_{C}, \end{array}$$

where *K* (in J.s${\,}^{-1}$.K${\,}^{-1}$) is the heat conductivity at $I_{C}=0$, *R* (in Ω) the electric resistance and α (in V.K${\,}^{-1}$) the Seebeck coefficient of the couple of materials involved in the TEG. We denote $\Delta T=T_{r}-T_{l}$ and $\Delta V=V_{r}-V_{l}$ respectively the temperature and voltage differences between the right and left contacts. This model is reviewed in Ref. [22] and is interpreted as follows: For α=0, the heat currents only have conductive (Fourier’s law) and dissipative (Joule’s law) contributions, proportional to ΔT and $I_{C}^{2}$, respectively. The Joule dissipation arises thanks to the electric current crossing the TEC; the corresponding electric work is fully dissipated into heat equally dumped into the left and right heat reservoirs, ensuring the validity of the first law ($i_{W}+i_{Ql}+i_{Qr}=0$). For α≠0, a thermoelectric coupling exists between the conductive and convective transport processes: in the EG mode, a part of electrons’ thermal energy is converted into electric work when they flow from hot to cold heat reservoirs.

**Figure 2:**
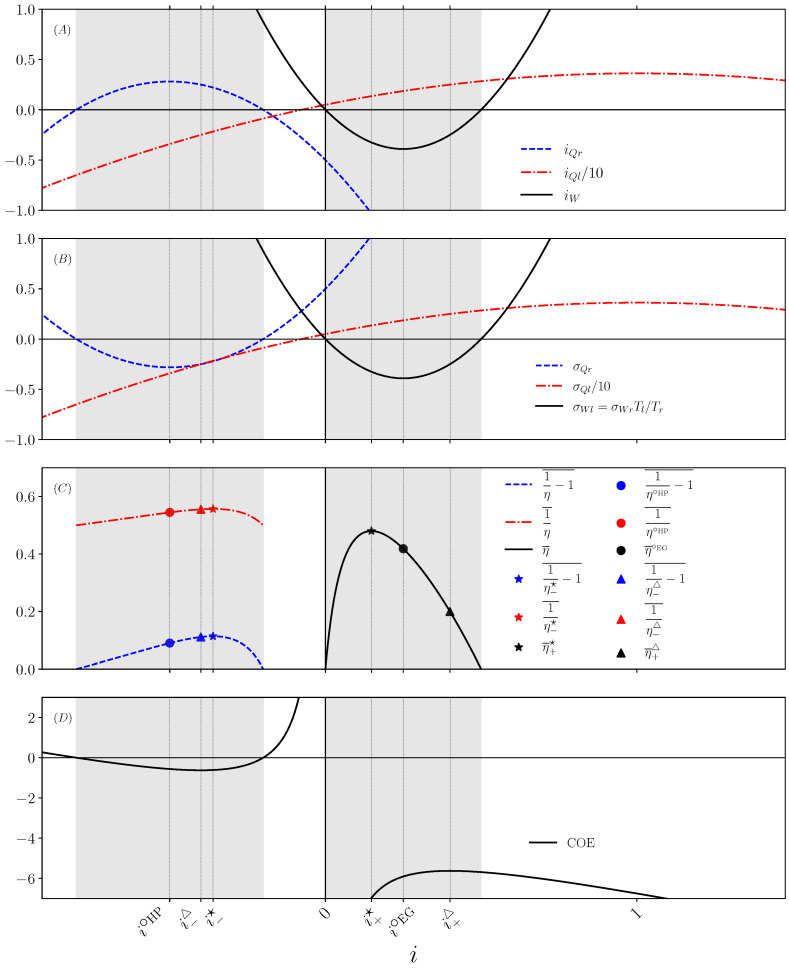
(**A**) Incoming currents: electric power $i_{W}$ (black solid line), heat currents incoming from the right $i_{Qr}$ (blue dashed line) and the left $i_{Ql}$ (red dot dashed line). (**B**) Partial EPR associated with each current (with same colors and line types). (**C**) Type II efficiencies for the CHP (blue dashed line), the HHP (red dot dashed line), and the EG (black solid line). Symbols label the three optima: filled circles for the working point of maximum extracted heat (i.e., Y=1 and maximum $i_{Qr}$) from the right (cold) reservoir in HP mode, or of maximum output power in EG mode (i.e., X=−1 and minimum $i_{W}$); filled stars for the working points of maximal efficiencies; filled triangles for the working point of extremal COE. (**D**) Cost of energy (COE). (All panels) Bottom horizontal axis indicates the dimensionless electric current *i*. Grey areas indicate the intervals of current compatible with the HP mode (on the left) or EG mode (on the right). Chosen parameters are $T_{r}=0.5$, $T_{l}=1$, K=1, R=1, and α=2.5. We use the ad hoc unit system in which $T_{r}$ sets the temperature unit, the unit Boltzmann constant $k_{B}=1$ sets the energy unit, the thermal conductivity sets the time unit via $KT_{r}/\left(k_{B}T_{r}\right)$, and the Seebeck coefficient α with the electric resistance *R* set the unit of the horizontal axis for dimensionless current via the ratio $4\alpha T_{r}/\left(5R\right)=1$.

To illustrate our discussion, the heat and work currents are provided as a function of the dimensionless electric current

(4) $$i\equiv \frac{RI_{C}}{\alpha T_{l}}$$

in the panel (A) of Figure 2.

Two decompositions of the EPR are relevant for the EG/HHP mode and for the CHP mode, respectively: $\sigma =\sigma_{Ql}+\sigma_{Wr}$, and $\sigma =\sigma_{Qr}+\sigma_{Wl}$, where

(HHP+EG 5) $$\begin{array}{cccc} \sigma_{Ql} & \equiv \left(\frac{1}{T_{r}}-\frac{1}{T_{l}}\right)i_{Ql},\; & \sigma_{Wr} & \equiv \frac{i_{W}}{T_{r}}, \end{array}$$

(CHP 5) $$\begin{array}{cccc} \sigma_{Qr} & =\left(\frac{1}{T_{l}}-\frac{1}{T_{r}}\right)i_{Qr},\; & \sigma_{Wl} & \equiv \frac{i_{W}}{T_{l}}. \end{array}$$

These partial EPRs as functions of *i* appear in panel (B) of Figure 2. The TEC operates in a non-trivial way when a current is opposed to its conjugated force in the EPR. This is sometimes called negative response in the framework of irreversible thermodynamics [24,25,26]. Then, among the two partial EPRs appearing in the total EPR, one is negative and one is positive. The operating modes of the TEC are defined as follows:

(EG 6) $$\begin{array}{cccccc} \sigma_{Wr} & <0\; & \mathrm{with} & \; & \sigma_{Ql} & >0, \end{array}$$

(HHP+CHP 6) $$\begin{array}{cccccc} \sigma_{Qr} & <0\; & \mathrm{with} & \; & \sigma_{Wl} & >0. \end{array}$$

This corresponds to the following intervals (shaded areas on Figure 2) for the electric current

(EG 7) $$\begin{array}{cc} I_{C} & \in ]0,-\frac{\alpha \Delta T}{R}[, \end{array}$$

(HHP+CHP 7) $$\begin{array}{cc} I_{C} & \in ]I_{C-};I_{C+}[, \end{array}$$

where we introduced

(8) $$I_{C\pm }=\frac{\alpha T_{r}}{R}\left(-1\pm \sqrt{1+\frac{2\Delta T}{ZT_{r}^{2}}}\right),$$

as the two electric currents for which $\sigma_{Qr}=0$. From Equation (8), it is clear that in our model the HP mode exists only if

(9) $$\Delta T>-\frac{ZT_{r}^{2}}{2},$$

meaning that too-high temperature differences compromise the existence of a HP mode.

For each operating mode and utility, following the terminology of Ref. [2], we define the type I efficiencies (or performance coefficients) as

(EG 10) $$\begin{array}{cc} \eta & \equiv -\frac{i_{W}}{i_{Ql}}, \end{array}$$

(HHP 10) $$\begin{array}{cc} \frac{1}{\eta } & \equiv -\frac{i_{Ql}}{i_{W}}, \end{array}$$

(CHP 10) $$\begin{array}{cc} \frac{1}{\eta }-1 & \equiv +\frac{i_{Qr}}{i_{W}}. \end{array}$$

The reversible (σ=0) value of these type I efficiencies are the Carnot efficiency $\eta_{C}=1-\frac{T_{r}}{T_{l}}$ in the EG mode, $1/\eta_{C}$ in the HHP mode, and $1/\eta_{C}-1$ in the CHP mode. The type II efficiencies are defined as the opposite of the ratio between the negative partial EPR in a given mode and the other positive partial EPR. It turns out that type II efficiencies correspond to type I efficiencies normalized to their reversible values. For instance for the EG mode, the type I efficiency is normalized by dividing it by the Carnot efficiency:

(EG 11) $$\begin{array}{cc} \bar{\eta } & \equiv -\frac{\sigma_{Wr}}{\sigma_{Ql}}=\frac{\eta }{\eta_{C}}, \end{array}$$

(HHP 11) $$\begin{array}{cc} \bar{\frac{1}{\eta }} & \equiv -\frac{\sigma_{Ql}}{\sigma_{Wr}}=\frac{\eta_{C}}{\eta }, \end{array}$$

(CHP 11) $$\begin{array}{cc} \bar{\frac{1}{\eta }-1} & \equiv -\frac{\sigma_{Qr}}{\sigma_{Wl}}=\frac{\frac{1}{\eta }-1}{\frac{1}{\eta_{C}}-1}. \end{array}$$

Therefore, the reversible value of type II efficiency is 1 in all cases. Panel (C) of Figure 2 indicates the variation in the type II efficiencies with the dimensionless electric current *i*.

Finally, we introduce the “cost of energy” as follows:

(12) $$\mathrm{COE}\equiv \frac{i_{Qr}}{I_{C}}.$$

In the EG mode, the TEC rejects heat in the cold reservoir ($i_{Qr}<0$). The COE therefore quantifies the amount of heat rejcted in the cold reservoir per unit of electrical current. In the HP mode, the TEC pumps heat from the cold reservoir ($i_{Qr}>0$). The COE therefore quantifies the amount of heat extracted from the cold reservoir per unit of electrical current. The COE is given as a function of the dimensionless electric current in panel (D) of Figure 2.

## 3. Three Optima of a TEC in EG Mode

We now turn to the problem of optimizing the TEC in the EG mode under fixed ΔT (or equivalently fixed $\eta_{C}$). We first determine

★ The maximal efficiency;
∘ The efficiency at maximum electric power;

▵

The efficiency at minimal waste.

As emphasized by the symbols in the above list, those optima are conveniently associated with stars, circles, and triangles for superscript in the formula below and to the same filled symbols on Figure 2. Then, we illustrate the power efficiency trade-off by showing that the efficiency is a bi-valued function of the electric power. We start by expressing the efficiency in terms of the following three dimensionless quantities: the Carnot efficiency $\eta_{C}$, the current i, and the figure of merit

(13) $$Z\bar{T}\equiv \frac{\alpha^{2}\bar{T}}{KR},\;\;\mathrm{with}\;\bar{T}\equiv \frac{T_{l}+T_{r}}{2}.$$

From Equation (EG10), we obtain the following:

(14) η=η(i)=ηC−iiηC2−ηC2ZT¯(+1−i2i.

Optimizing the efficiency with respect to the dimensionless current *i* leads to the following quadratic equation:

(15) $$\begin{array}{ccc} 0 & = & \left(1-\frac{\eta_{C}}{2}\right)i^{2}+\frac{\eta_{C}\left(2-\eta_{C}\right)}{Z\bar{T}}i-\frac{\eta_{C}^{2}\left(2-\eta_{C}\right)}{2Z\bar{T}}, \end{array}$$

which yields two solutions

(16) $$i_{\pm }^{★}=\frac{\eta_{C}}{Z\bar{T}}\left(-1\pm \sqrt{1+Z\bar{T}}\right)=\frac{\eta_{C}}{1\pm \sqrt{1+Z\bar{T}}}.$$

Before simplifying dη/di=0 into the quadratic Equation (15), it can be used to simplify the efficiency at the optimal dimensionless current as follows:

(17) $$\eta_{\pm }^{★}\equiv \eta \left(i_{\pm }^{★}\right)=\frac{\eta_{C}-2i_{\pm }^{★}}{1-i_{\pm }^{★}}.$$

Inserting Equation (16) into Equation (17), we obtain:

(18) $${\bar{\eta }}_{\pm }^{★}=\frac{\eta_{\pm }^{★}}{\eta_{C}}=\frac{-1\pm \sqrt{1+Z\bar{T}}}{1-\eta_{C}\pm \sqrt{1+Z\bar{T}}}.$$

In the EG mode, the maximum efficiency is ${\bar{\eta }}_{+}^{★}\in \left[0,1\right]$ obtained for the dimensionless current $i_{+}^{★}$. In the close-to-equilibrium limit $\eta_{C}\to 0$, we recover the maximum efficiency given in Equation (85) of Ref. [27]. The limit of strong coupling ($Z\bar{T}\to \infty$) leads to the Carnot efficiency, corresponding to $\bar{\eta }\to 1$.

Optimizing the output electric power (positive in EG mode),

(19) $$-i_{W}=\frac{\alpha^{2}T_{l}^{2}}{R}\left(\eta_{c}-i\right)i>0$$

with respect to *i* leads to the efficiency at maximum power

(20) $${\bar{\eta }}^{\circ_{\mathrm{EG}}}\equiv \eta \left(i^{\circ_{\mathrm{EG}}}\right)=\frac{1}{\frac{1-\eta_{C}/2}{4Z\bar{T}}+2-\frac{\eta_{C}}{2}},\;\mathrm{for}\;\;i^{\circ_{\mathrm{EG}}}=\eta_{C}/2,$$

associated to an output power

(21) $$-i_{W}^{\circ_{\mathrm{EG}}}=\frac{\alpha^{2}T_{l}^{2}\eta_{C}^{2}}{4R}.$$

The limit of strong coupling produces

(22) $$\lim_{Z\bar{T}\to \infty }{\bar{\eta }}^{\circ_{\mathrm{EG}}}=\frac{1}{2-\eta_{C}/2}≃\frac{1}{2}+\frac{1}{8}\eta_{C}+o\left(\eta_{C}\right),$$

where we made a close to equilibrium expansion in the last equality, recovering the universal expression of efficiency at maximum power [6].

For the last optimal point, we consider the cost of energy of Equation (12) rewritten as follows:

(23) $$\mathrm{COE}=-\frac{\alpha \eta_{C}}{Zi}-\alpha T_{r}-\frac{\alpha T_{l}}{2}i.$$

Its optimization with respect to *i* leads to the efficiency at the optimal wasted heat (per unit of electric current)

(24) $${\bar{\eta }}_{\pm }^{{\,}_{▵}}=1-i_{\pm }^{{\,}_{▵}}/\eta_{C},\;\mathrm{for}\;\;i_{\pm }^{{\,}_{▵}}=\pm \sqrt{\frac{\eta_{C}\left(2-\eta_{C}\right)}{Z\bar{T}}}.$$

**Figure 3:**
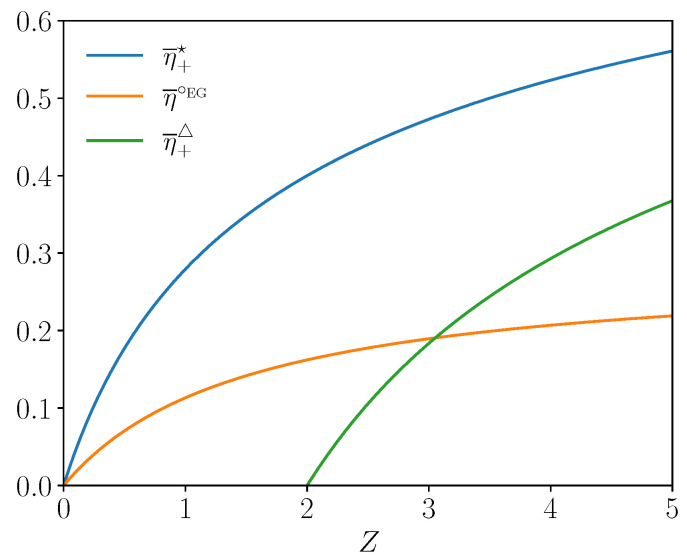
EG efficiency optima as a function of *Z* for $\eta_{C}=1/2$ and $\bar{T}=3/2$.

Due to the positivity of the electric current in the EG mode, the optimal applies for $i_{+}^{{\,}_{▵}}$ and the type II efficiency at minimal wasted heat is ${\bar{\eta }}_{+}^{{\,}_{▵}}$. We notice that the EG interval of current in Equation (EG7) is written for dimensionless current $i\in [0,\eta_{C}]$. Hence, it can happen that there is no optimal working point with minimal wasted heat in the EG mode if $i_{+}^{{\,}_{▵}}>\eta_{C}$ (most likely for small $Z\bar{T}$). We summarize the three optima of the EG efficiency derived above as a function of *Z* for a fixed Carnot efficiency in Figure 3. We recover that the efficiencies associated to the three optima grow with *Z* and converge asymptotically to a maximum value. For parameters of Figure 3, we notice that the EG efficiency at maximal COE changes of sign at Z=2 meaning that this optimal working point does not exist below this threshold.

We end this section by analyzing the power efficiency trade-off in the EG mode. Since both efficiency and power are functions of the electric current, one can draw the efficiency–power relation as a parametric plot. We derive an analytical expression of efficiency as a function of the power to maximum power ratio below:

(25) $$X\equiv -\frac{i_{W}}{i_{W}^{\circ_{\mathrm{EG}}}}=\frac{4(i-\eta_{C})i}{\eta_{C}^{2}}\geq -1\;\;\mathrm{for}\;i\in \left[0,\eta_{C}\right].$$

Since, in the EG mode, $i_{W}^{\circ_{\mathrm{EG}}}<0$ and $i_{W}<0$, *X* belongs to $\left[-1,0\right]$. The dimensionless current as a function of the power to maximum power ratio is expressed as follows:

(26) $$i_{\pm }=\frac{\eta_{C}}{2}\chi_{\pm },\;\;\mathrm{with}\;\chi_{\pm }\equiv 1\pm \sqrt{1+X}.$$

**Figure 4:**
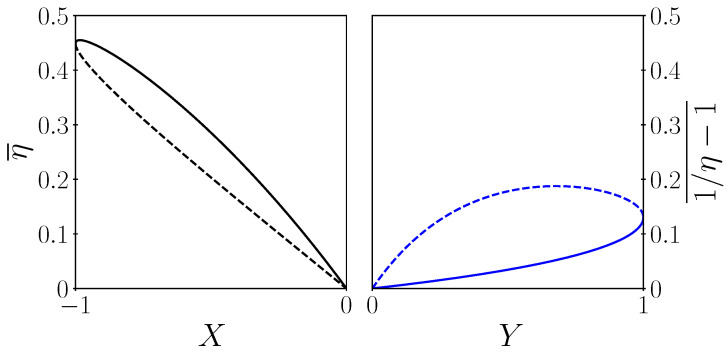
Type II efficiencies $\bar{\eta }\left(X\right)$ (**left** panel) and $\bar{\frac{1}{\eta }-1}\left(Y\right)$ (**right** panel) of the EG and CHP as a function of *X* and *Y*, respectively, i.e., the electric and thermal power to maximum power ratios. Solid (+ case) and dashed lines (− case) are for the two branches (± signs) in Equations (27), (37) and (38). Parameters are $T_{l}=1$, $T_{r}=0.5$, K=1, R=1, α=2.5.

Its use in the type II efficiency gives

(27) $$\bar{\eta }=\frac{\left(1-\frac{\chi_{\pm }}{2}\right)\frac{\chi_{\pm }}{2}}{\frac{2-\eta_{C}}{2Z\bar{T}}+\left(1-\frac{\chi_{\pm }\eta_{C}}{4}\right)\frac{\chi_{\pm }}{2}},$$

that is represented in the left panel of Figure 4. Using $i_{+}^{★}$ of Equation (16) in Equation (25), one finds that the power ratio at maximum efficiency is

(28) $$X^{★}=-4\frac{\sqrt{1+Z\bar{T}}}{{\left(1+\sqrt{1+Z\bar{T}}\right)}^{2}}.$$

The left panel of Figure 4 displays the typical lobe shape of power–efficiency curves, with two particular points: the maximum efficiency point at $\left(X^{★},{\bar{\eta }}_{+}^{★}\right)$ and the efficiency at maximum power point at $\left(-1,{\bar{\eta }}^{\circ_{\mathrm{EG}}}\right)$.

It is important to note that the relative position of the three operating points in Figure 2 is not general for all thermodynamic machines. To be more precise, as the current increases, the maximum efficiency is always reached before the maximum power, regardless of the thermodynamic machine. The location of the maximum of COE is different. For example, in the case of the modeling of biological systems such as muscles [13,21], the minimum COE point is between the maximum efficiency and maximum power points. This is because, in these systems, the viscous dissipation term *R* is very small, which is clearly never the case in thermoelectric systems. Note that in this work, the optimal COE is a maximum since our sign convention, see Figure 1, is different from the one chosen in [13].

In addition, we would like to mention that the literature on the optimization of TEC has largely focused on maximum electricity production, and more rarely on optimizing efficiency. Indeed, most applications involve the use of waste heat, which does not represent any cost. In the rare cases where this heat has to be produced, and, therefore, paid for, efficiency naturally becomes the objective to be pursued. The case of the optimization of the COE seems to be excluded from all these considerations. However, it becomes central in all situations where the cold source is a temperature reservoir of limited size, or if access to this source is limited by a poor heat exchanger. In this case, the question of minimizing the thermal power rejected becomes the main argument. This is the case, for example, with all mobile applications, including thermoelectric conversion in the automotive sector, where this problem has proven to be the most significant. It is also the case with radioisotope thermoelectric generators (RTGs), where the size of the radiative panels is limited by space constraints.

## 4. Three Optima of a TEC in HP Mode

We now turn to the problem of optimizing the TEC in HP mode. The differences between the two utilities are not relevant, except that the heat injected into the hot reservoir has no maximum in this mode, while the extracted heat from the cold reservoir does. Given that EG and HP efficiencies are inverse of each other (see Equations (EG10)–(HHP10)), we use the results of Section 3 to determine the maximal efficiency, the efficiency at maximum thermal power, and the efficiency at minimum COE under fixed ΔT (or equivalently fixed $\eta_{C}$). We keep the notations associated to those optima, respectively: stars, circles, and triangles for superscript in the formula below associated with the same filled symbols on Figure 2. Then, we illustrate the power efficiency trade-off by showing that the efficiency is a bi-valued function of the thermal power.

Optimizing the efficiency 1/η for the HHP (or 1/η−1 for the CHP) with respect to the dimensionless current *i* follows directly from the extrema of η for the EG. An exact computation software can show that the remaining root $i_{-}^{★}$ of Equation (15) belongs to the interval of the electric current compatible with the HP mode as follows:

(29) $$\frac{RI_{C-}}{\alpha T_{l}}\leq i_{-}^{★}\leq \frac{RI_{C+}}{\alpha T_{l}}<0.$$

From this dimensionless current, the efficiency $\eta_{-}^{★}$ of Equation (17) can be used to write the maximum performance coefficient of the HP (type I efficiency)

(30) $$\frac{1}{\eta_{-}^{★}}-1=\frac{1-\eta_{C}-i_{-}^{★}}{\eta_{C}-2i_{-}^{★}},$$

or after normalizing by $1/\eta_{C}-1$

(31) $$\bar{\frac{1}{\eta_{-}^{★}}-1}=\frac{1-\frac{\eta_{C}}{Z\bar{T}\left(1-\eta_{C}\right)}\left(1+\sqrt{1+Z\bar{T}}\right)}{1+\frac{2}{Z\bar{T}}\left(1+\sqrt{1+Z\bar{T}}\right)},$$

for the type II efficiency.

The maximum of the output thermal power $i_{Qr}$ of the CHP is

(32) $$i_{Qr}^{\circ_{\mathrm{HP}}}=K\Delta T+\frac{\alpha^{2}T_{l}^{2}}{2R}{\left(1-\eta_{C}\right)}^{2}.$$

It is achieved for the optimal dimensionless current $i^{\circ_{\mathrm{HP}}}=\eta_{c}-1$. This current used in Equation (14) defines $\eta^{\circ_{\mathrm{HP}}}\equiv \eta \left(i^{\circ_{\mathrm{HP}}}\right)$, leading to a performance coefficient at the maximum thermal power (i.e., type I efficiency)

(33) $$\frac{1}{\eta^{\circ_{\mathrm{HP}}}}-1=\frac{\eta_{C}\left(\eta_{C}-2\right)}{2Z\bar{T}\left(1-\eta_{C}\right)}+\frac{1-\eta_{C}}{2}$$

or after normalizing by $1/\eta_{C}-1$

(34) $$\bar{\frac{1}{\eta^{\circ_{\mathrm{HP}}}}-1}=\frac{\eta_{C}^{2}\left(\eta_{C}-2\right)}{2Z\bar{T}{\left(1-\eta_{C}\right)}^{2}}+\frac{\eta_{C}}{2}$$

for the type II efficiency.

**Figure 5:**
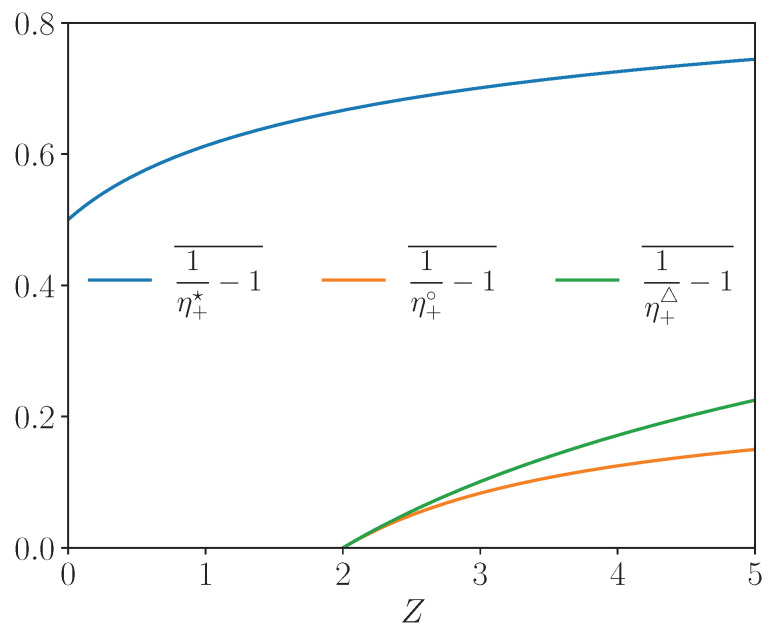
CHP efficiency optima as a function of the *Z* for $\eta_{C}=1/2$ and $\bar{T}=3/2$.

For the last optimal point, the negative current $i_{-}^{{\,}_{▵}}$ minimizes the COE. This corresponds to a minimum heat extraction from the cold reservoir per unit of electric current. This dimensionless current used in Equation (14) defines $\eta_{-}^{{\,}_{▵}}\equiv \eta \left(i_{-}^{{\,}_{▵}}\right)$, leading to a performance coefficient at minimum COE (type I efficiency)

(35) $$\frac{1}{\eta_{-}^{{\,}_{▵}}}-1=\frac{1}{\eta_{C}-i_{-}^{{\,}_{▵}}}-1,$$

or after normalizing by $1/\eta_{C}-1$

(36) $$\bar{\frac{1}{\eta_{-}^{{\,}_{▵}}}-1}=\frac{1+\frac{i_{-}^{{\,}_{▵}}}{1-\eta_{C}}}{1-\frac{i_{-}^{{\,}_{▵}}}{\eta_{C}}},$$

for the type II efficiency. We summarize the three optima of the CHP efficiency derived above as a function of the figure of merit *Z* for a fixed Carnot efficiency in Figure 5. Here again, the efficiencies associated to the three optima grow with *Z* and converge asymptotically to a maximum value. For parameters of Figure 5, the CHP efficiency at maximum thermal power and the one at minimum COE change of sign at Z=2 meaning that these optima do not exist below this threshold.

We end this section by analyzing the power efficiency trade-off in the HP mode. The ratio between the thermal power and the maximum thermal power is

(37) $$Y\equiv \frac{i_{Qr}}{i_{Qr}^{\circ_{\mathrm{HP}}}}=\frac{\eta_{C}+\frac{2Z\bar{T}}{2-\eta_{C}}\left(1-\eta_{C}+i/2\right)i}{\eta_{C}-\frac{Z\bar{T}{\left(1-\eta_{C}\right)}^{2}}{2-\eta_{C}}}\in \left[0,1\right].$$

The dimensionless current as a function of this thermal power ratio is

(38) $$i_{\pm }=\eta_{C}-1\pm \sqrt{\frac{1-Y}{Z\bar{T}}\left[\left(1+Z\bar{T}\right)\eta_{C}\left(\eta_{C}-2\right)+Z\bar{T}\right]}.$$

We remark that $i_{\pm }$ for Y=0 leads to the boundaries of the HP mode $RI_{C\pm }/\left(\alpha T_{l}\right)$ (for dimensionless current) and $i^{\circ_{\mathrm{HP}}}=\eta_{C}-1$ for Y=1 as expected. Using the above current as a function of the thermal-power-to-max-thermal-power ratio in the type II efficiency, $\bar{1/\eta -1}$ for η of Equation (14) leads to an explicit power efficiency relation that is shown on the right panel of Figure 4. As in the EG case, this figure displays the typical lobe shape of power–efficiency curves, with its two particular points: the maximum efficiency point at $\left(Y^{★},\bar{\frac{1}{\eta_{-}^{★}}-1}\right)$ and the efficiency at maximal thermal power point at $\left(1,\bar{\frac{1}{\eta^{\circ_{\mathrm{HP}}}}-1}\right)$. We defined $Y^{★}\equiv Y\left(i_{-}^{★}\right)$ using Equations (16) and (37). As in the case of the EG operation mode, the question of the relevance of one of the three optima compared to another is crucial in the case of an HP. It depends on the application and above all on the nature of the sources and their coupling to the machine. Contrary to the EG case, the power supplied to the system always has a cost that is invoiced. This explains why, in operation, the maximum efficiency point is more sought after than the maximum pumping power point. We note that the COE curve in HP mode shows that the COE minimum point is close to that of maximum efficiency. The maximum efficiency point does not quite coincide with the maximum heat rejection point. There is therefore a fairly subtle adjustment that can be made to the incident electrical power to optimise the rejection of heat.

## 5. Conclusions

The optimization of a nonlinear converter in all its possible operating modes is, in general, challenging but is possible analytically for a TEC satisfying the CPM. We have shown that, in order to optimize the operation modes of such a TEC, it suffices to compute the optima of the EG efficiency. Indeed, the HP efficiencies (heating or cooling) are equal to the inverse of the EG efficiency (up to a constant for the CHP). This deeply simplifies the search of the TEC’s optimal working points. Accordingly, we can conclude that for all operating modes and whatever the chosen optimum (efficiency, power, or wasted heat), the increase in the figure of merit ZT always improves the TEC’s performance. Beyond the optimal working points we have provided in this work, the analytical efficiency–power trade-off for both the EG and CHP operation modes. An interesting perspective would be investigating whether these ideas on device optimization apply to more complex models of TECs or even to any kind of converter. The fact that efficiency is a bi-valued function of the output power is rather universal in thermodynamic conversion (and even beyond in economics, numerical computing, etc.); the two branches correspond to a upper and a lower values of efficiency for the same power output. Disruptive events may trigger switch from the upper to the lower branch. Understanding these events are fundamental for the safety of power plants, e.g., for hydroelectric power stations, since such switches are synonymous of a sudden increase in power dissipation that can lead to operational accidents. Finally, the biologically inspired optimum of conversion with “minimal waste” might also be of valuable use in some applications when one targets sobriety. Beyond thermoelectricity, but still for thermal converters, the concept of COE gives an interpretation of the cooling efficiency outside of the cooling range (i.e., in the EG operation mode). Summer electric generation during heat waves is optimal for the lowest possible river heating per unit of output electric power.

## Data Availability

The original contributions presented in this study are included in the article. Further inquiries can be directed to the corresponding author.
